# Exploring factors influencing autistic children and young people’s access to dental care in Southwest England: a qualitative study of children, parents, and dental professionals

**DOI:** 10.1186/s12903-026-08068-1

**Published:** 2026-03-10

**Authors:** Jo Erwin, Sarah Neill, Tara Vassallo, Robert Witton, Louise Peters, Isaac Vassallo, Abigail Nelder, Martha Paisi

**Affiliations:** 1 Peninsula Dental School, University of Plymouth, Plymouth, UK; 2 School of Nursing and Midwifery, University of Plymouth, Plymouth, UK; 3 Plymouth Institute of Education, University of Plymouth, Plymouth, UK; 4 National Autistic Society - Plymouth & District Branch, Plymouth, UK; 5 Peninsula Dental Social Enterprise, Plymouth, UK; 6 Artist and Community worker, Cornwall, UK; 7 School of Engineering, Computing and Mathematics, University of Plymouth, Plymouth, UK

**Keywords:** Autistic, Children and Young People, Dental care, Access

## Abstract

**Background:**

Autistic children and young people (CYP) experience inequalities in oral health. They are at higher risk of dental caries and periodontal disease and are more likely to have teeth extracted under general anaesthetic than their non-autistic peers. Poor oral health among autistic CYP results from multiple factors, including sensory sensitivities, communication differences, and difficulties accessing appropriate dental care. This qualitative study explored factors influencing autistic CYP’s access to dental care from the perspective of CYP, parents/carers, and dental health professionals (DHPs).

**Methods:**

A qualitative design using semi-structured interviews was adopted. Purposive sampling was used to recruit autistic CYP, parents/carers, and DHPs in Southwest England. Data were collected online, by telephone, or face-to-face according to participant preference and accessibility needs. Interviews followed co-produced topic guides covering daily oral care, challenges and enablers, dental visit experiences, and views on improving support for autistic CYP. Audio recordings were transcribed verbatim and analysed thematically using inductive and deductive coding within NVivo 12.

**Results:**

Nineteen autistic CYP (aged 5–18 years), 20 parents/carers, and 16 DHPs were interviewed. Analysis revealed a complex interplay of individual, provider, and organisational factors shaping access to dental care. Sensory, communicative, and systemic challenges intersected, affecting how care was experienced, delivered, and sustained. Three overarching domains were identified: (1) CYP-related factors, including sensory sensitivities, communication differences, anxiety, and transition challenges; (2) provider-related factors, such as awareness of autism, rapport, flexibility, and time constraints; and (3) organisational influences, including NHS contract limitations, poor information sharing, and the need for autism-focused training.

**Conclusions:**

Autistic CYP face distinct barriers to dental care, compounded during transition to adulthood. NHS dental professionals often lack the time, flexibility, and continuity required for individualised support. Enhancing information-sharing, referral pathways, integrated care, and autism-informed training could improve equity and accessibility. Findings highlight the need for system- and professional-level interventions to ensure dental care is inclusive and responsive to autistic CYP.

**Supplementary Information:**

The online version contains supplementary material available at 10.1186/s12903-026-08068-1.

## Background

Poor oral health (OH) is largely preventable yet remains a serious problem among children in the UK [1], with implications for eating, sleeping, school attendance and long-term wellbeing [2]. Autistic children and young people (CYP) are disproportionately affected, exhibiting higher prevalence of dental caries and periodontal problems, and are more likely to require tooth extractions under general anaesthesia than their non-autistic peers [3]. The oral health challenges experienced by autistic CYP are multifactorial and may relate to sensory sensitivities, dietary preferences, oral hygiene routines, and anxiety within dental settings [4]. The issue is further compounded by difficulties accessing suitable dental care and limited professional awareness of autism-specific needs [5]. Recent qualitative research by Chauhan et al. [6] has vividly illustrated autistic children’s experiences as “an explosion in the mouth,” highlighting how sensory overload and communication difficulties combine to make dental visits distressing.

Access to healthcare, including dental care, is a multidimensional construct shaped by the interplay between service characteristics and population needs [7–12]. For autistic CYP, these dimensions are influenced by sensory processing differences, communication preferences, and the responsiveness of dental systems [5]. Existing frameworks of healthcare access [10, 11] are rarely applied to neurodivergent populations, limiting understanding of how structural and interpersonal barriers interact.

While previous studies have examined parental perspectives on dental access for autistic children [13], few have included the direct voices of autistic CYP themselves [5], and none has integrated perspectives from multiple stakeholders. Engaging autistic CYP as active participants offers valuable insight into their perspectives and experiences, helping to inform more inclusive dental care. This study addresses this gap by exploring the factors influencing autistic CYP’s access to dental care from the perspectives of CYP, their parents/carers, and dental health professionals (DHPs).

The aim of this qualitative study was to explore the factors influencing autistic CYP’s access to, and experiences of, dental care in Southwest England.

## Methods

The methods used in this project have been previously described [14].

### Study design

This study adopted a qualitative design using semi-structured interviews to explore the experiences of autistic CYP, their parents or carers, and DHPs in Southwest England. Interviews were chosen to capture participants’ perspectives and experiences in depth and to allow flexibility in communication style and environment.

### Patient and public involvement

The research was co-produced with a Research Advisory Group comprising four autistic CYP (aged 9–17 years) and their parents. They advised on study design, recruitment strategies, and topic-guide development, and contributed to interpreting the findings and manuscript writing.

### Research team and reflexivity

Interviews were conducted by JE, a female post-doctoral Research Fellow in Public Health Dentistry trained in qualitative research. She has previous experience interviewing autistic children and personal insight through a close family connection to autism. Other team members (SN, MP, RW) are experienced qualitative researchers in child health, dentistry and public health. Reflexive discussion occurred throughout analysis to consider how researchers’ disciplinary backgrounds and perspectives might shape interpretation.

### Sampling strategy

A purposive sampling approach was used to ensure diversity of age, gender, and role (i.e., DHPs) across participant groups. Prior studies indicate that rich thematic data is typically achieved with 15–20 interviews per group. Consequently, we aimed to recruit 20 CYP, 20 parents/carers, and 15–20 DHPs [13, 15]. Sample sizes were continually evaluated against the concept of information power, that is, the adequacy of the data to address the research question, and interviews were conducted until participant responses were judged sufficient to provide comprehensive insight into the topic [16, 17].

### Eligibility criteria

Participants were eligible if they met one of the following criteria:

1. Autistic CYP aged 5–19 years, living and/or attending school in Southwest England. Both a confirmed autism diagnosis and being on the diagnostic pathway were acceptable for inclusion.
2. Parents or carers of autistic CYP living and/or attending school in Southwest England.
3. DHPs providing care for autistic CYP in the region. All members of the dental team were eligible, including dentists, dental hygienists, dental nurses, oral health educators, and reception staff.

### Ethical approval and considerations

This study received ethical approval from the University of Plymouth, Faculty of Health Research Ethics and Integrity Committee (approval number 2022-3029-2777). The research was conducted in full compliance with the principles of the Declaration of Helsinki.

Informed consent or assent was obtained from all participants. Age- and ability-appropriate information sheets, consent, and assent forms were developed using simple language and visual aids to support understanding. Consent procedures were tailored to participants’ age and capacity.

### Protection from harm

JE, the interviewing researcher, remained alert to any signs of distress or withdrawal during interviews, which could be subtle and individual. During initial “getting to know you” sessions, she discussed with the CYP and/or parent/carer how to recognise signs of discomfort or anxiety and strategies to create a supportive environment.

If a CYP indicated a wish to stop, or the parent/carer felt the child was becoming distressed, the interview was gently brought to a close. These procedures were reinforced throughout data collection to prioritise participant wellbeing and ensure ongoing assent or consent.

JE had DBS clearance, safeguarding training, and training in informed paediatric consent, ensuring that all interactions were conducted safely and ethically.

### Recruitment and consent procedures

Recruitment was facilitated through local schools, autism support organisations, and professional dental networks. Information about the study was shared via newsletters, social media groups, and professional bulletins. Interested participants could contact the research team directly or through partner organisations.

For autistic CYP and their parents/carers, those wishing to learn more could respond via their school or supporting organisation, or by contacting the researcher directly. The researcher then provided an information sheet and consent form to those who expressed interest.

For DHPs, interested individuals were invited to visit the study website to access the participant information and complete an online consent form, or alternatively, contact the researcher directly for further details.

Parents or legal guardians provided consent for their children to be approached about participation.

- For children aged 5–10 years (based on chronological age or equivalent level of understanding), consent was obtained from the parent or legal guardian, and, where possible, written assent was also sought from the child. Verbal assent was reconfirmed at the start of each interview.
- For CYP aged 11–17 years assessed to have Gillick competence [18], written consent was obtained directly from the young person, with competence assessed by their parent or guardian. For those not deemed Gillick competent, parental or guardian consent was obtained, together with the written and/or verbal assent of the child or young person.
- Written informed consent was obtained from all participants aged 18–19 years, as well as from all adult participants, including parents/carers and DPHs.

### Topic guides

Four semi-structured interview guides were co-developed to explore oral health and dental care experiences among autistic CYP, their parents/carers, and dental professionals (Supplementary File 1). Those for CYP and parents/carers were developed together with members of the Research Advisory Group and were piloted with members from a local youth club designed for neurodivergent CYP (4 Me and My Friends) and their parents/carers. The DHP interview guide was developed with members of the research team and piloted with professional colleagues not connected with this study.

Each guide followed three core sections:

- Introduction: Built rapport and collected contextual information (e.g., daily routines, autism diagnosis, participant role).
- Oral health at home: Explored toothbrushing routines, challenges, supports, and enablers.
- Dental care experiences: Investigated dental visits, barriers and facilitators, effective dental team qualities, and recommendations for improving access and comfort.

Guides were tailored to each group: young children focused on routine and enjoyment of care; older children and young people reflected on independence and coping strategies; parents/carers focused on managing oral care and navigating services; dental professionals focused on experiences, challenges, confidence, and training needs.

### Data collection

Data were collected between July 2022 and January 2023. Interviews lasted 25–70 min and were audio-recorded with permission. Interviews were conducted online, by telephone, or face-to-face according to participant preference and accessibility needs. Offering multiple modes allowed participants to choose the most comfortable and least anxiety-provoking environment.

Interviews with CYP and their parents/carers were conducted online or face-to-face. JE met with participants before the interview to introduce herself and build rapport with the CYP, sharing information about her background, interests, and hobbies, and asking about their daily lives, hobbies, likes and dislikes, and communication preferences.

For autistic CYP, the option to engage from familiar settings (e.g., home) often enhanced communication. Recognising developmental and personality differences, CYP were given the choice of being interviewed alone or with a parent/carer present. These approaches addressed potential challenges in communication and engagement among younger participants, consistent with evidence that cognitive, developmental, and personality factors can affect children’s participation in research [19].

Interviews with DHPs were held online or by telephone. Field notes captured contextual information and researcher reflections. No repeat interviews were held. Transcripts were produced verbatim in English, anonymised, and returned to participants for optional checking; three participants provided minor corrections.

### Data analysis

Data were analysed using a hybrid reflexive thematic approach [20–22] within NVivo 12. This approach combined deductive and inductive coding. Deductive coding was guided by the study aims and research questions, providing a structured lens to identify anticipated patterns and areas of interest. Inductive coding was data-driven, allowing themes and patterns to emerge organically from participants’ accounts, ensuring new insights were captured.

Initial coding was conducted by JE and SN and refined through iterative discussion with the research team (JE, MP, SN, RW), enabling identification of areas for exploration in subsequent interviews. Codes were then organised into broader themes to capture patterns of meaning across the dataset. Themes were reviewed and defined collaboratively, reaching consensus to ensure robust and reflexive interpretation of the findings.

The study adhered to COREQ guidelines [23] and employed multiple strategies to ensure rigour and enhance the trustworthiness of the findings. Credibility was supported through member checking, data triangulation of perspectives across CYP, parents, and DHPs, and illustrative data quotes; transferability was addressed through thick descriptions of the study setting and participants; dependability and auditability were ensured via a detailed audit trail and researcher triangulation; and confirmability was strengthened through reflexive journaling and auditing procedures [24, 25].

## Findings

A total of 55 participants were interviewed: 19 autistic CYP (aged 5–18 years), 20 parents/carers, and 16 DHPs.

Tables 1 and 2 show the characteristics of the interviewees.

**Table 1:** Characteristics of DHP interviewees

ID	Profession
DID1	Dental Hygienist
DID2	Dental hygienist
DID3	Specialist in Paediatric Dentistry
DID4	Community Dentist
DID5	Specialist Trainee in Special Care Dentistry
DID6	General Dental Practitioner
DID7	General Dental Practitioner
DID8	Dental Nurse /Oral Health Educator
DID9	Dental Nurse / Oral Health Educator
DID10	Specialist in Special Care Dentistry
DID11	Dental Nurse / Oral Health Educator
DID12	Community Dentist
DID13	Community Dentist
DID14	General Dental Practitioner
DID15	General Dental Practitioner
DID16	Consultant in Paediatric Dentistry

*DID *Dental Identifier

**Table 2:** Characteristics children/young people and parent/carer interviewees

ID	Parent/Carer	Child (age group in years)
ID1 & ID2	Mother	Daughter (5–9)
ID3 & ID4	Mother	Son (15–19)
ID5 & ID6	Mother	Daughter (15–19)
ID7 & ID8	Mother	Son (15–19)
ID9 & ID10	Mother	Son (10–14)
ID11 & ID12	Grandmother	Grandson (10–14)
ID13 & ID14	Mother	Son (15–19)
ID15	Mother	(Son 10–14: did not take part in research)
ID16 & ID17	Mother	Daughter (10–14)
ID18 & ID19	Mother	Son (10–14)
ID20	Father	(Son 5–9: did not take part in research)
ID21	Mother	(Daughter 10–15: did not take part in research)
ID22 & ID23	Mother	Son (10–14)
ID24 & ID25	Mother	Daughter (10–14)
ID26, ID31, ID37	Mother	Son (15–19), Son (10–14)
ID27, ID28, ID29, ID30	Mother, Father	Daughter (10–15), Son (5–9)
ID32 & ID33	Mother	Daughter (5–9)
ID34, ID35, ID36	Mother, Father	Daughter (10–14)
ID38 & ID39	Did not take part in research	Son (5–9), Son (5–9)

*ID *Identifier

Analysis generated five interrelated themes illustrating the complex interplay between individual, relational, and organisational factors influencing access to dental care for autistic CYP.

Table 3 presents an overview of themes and subthemes identified in the data.

**Table 3:** Overview of themes and subthemes

1.Autistic CYP’s experience of going to the dentist
1.1 Positive and negative experiences
1.2 Impact of negative experience on future access
2.Access to dental care by autistic CYP
2.1 Service limitations
2.2 Lack of choice and its consequences
2.3 Private care as a partial solution
3.CYP-related factors influencing dental experience and access
3.1 Sensory sensitivities
3.2 Changes in routine and unfamiliar environments
3.3 Anxiety and fear of the dentist
3.4 Transition to adulthood and executive functioning
4.Provider-related factors affecting access
4.1 Awareness and understanding of autism among DHP
4.2 Communication
4.3 Trust and rapport
4.4 Parent/carer advocacy
4.5 Flexibility and time constraints
5.Organisational changes and training to improve access to dental care for autistic CYP
5.1 Improving access
5.2 Sharing information
5.3 Training needs
6. Key messages for dental students

*CYP *Children and Young People, *DHP *Dental Health Professionals

**Fig. 1:**
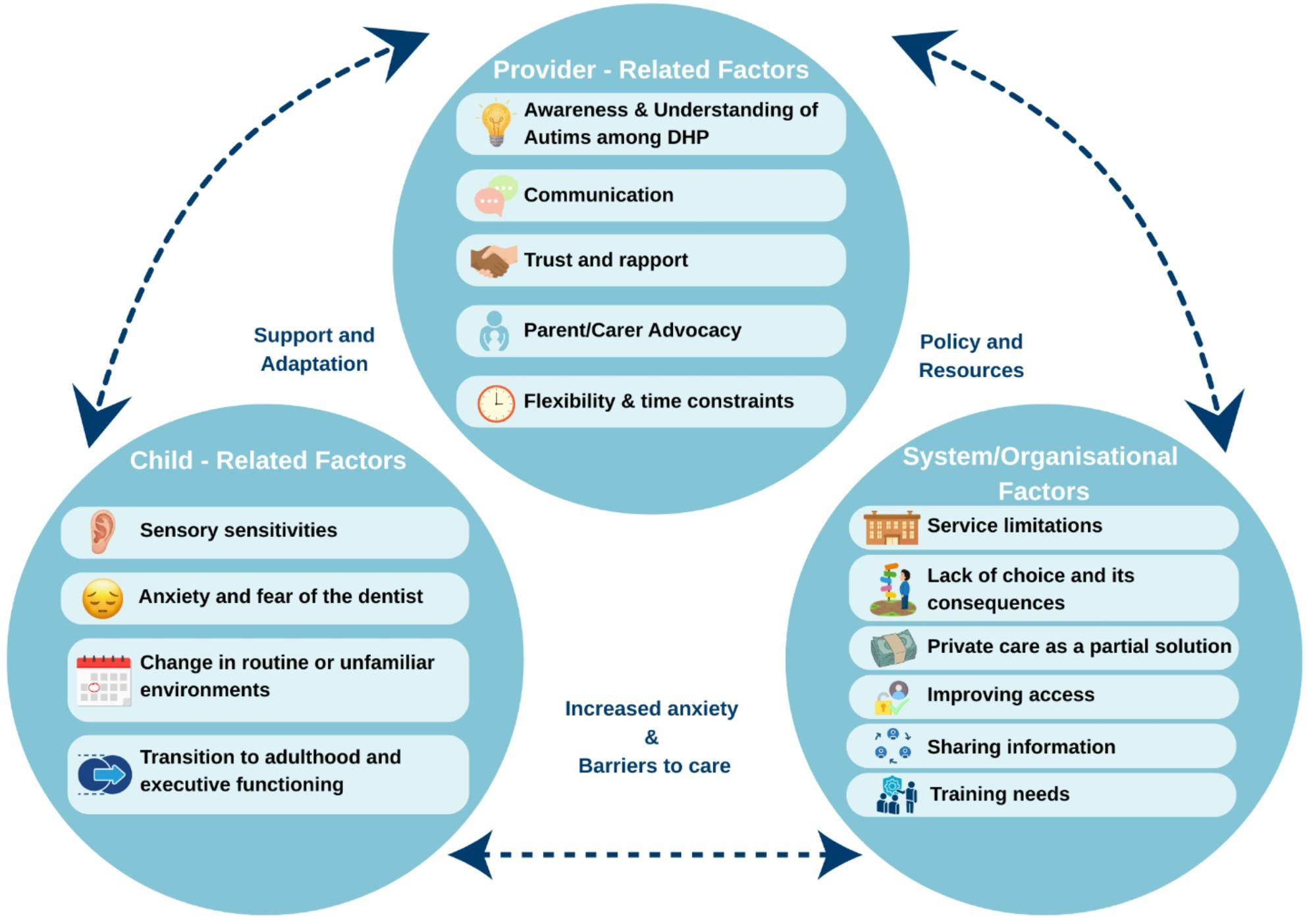
Factors impacting dental care access for autistic children

Figure 1 illustrates the overarching domains, subthemes and their interactions.

Below the themes and subthemes are described with illustrative quotes from participants, age range in years shown in brackets.

### Theme 1: autistic CYP’s experience of going to the dentist

#### Positive and negative experiences

Autistic CYP and their parents/carers reported a wide range of experiences during dental visits, showing how interactions with dental staff, routines, and sensory environments shaped their perceptions. Positive experiences were characterised by patience, empathy, and predictable routines:

*“He’s [dentist] really*,* really good. He’s really*,* really patient… the dentist did do a social story and everything for [ID31]… picture of the mirror and gloves and the lady and the nurse. That has helped… the last two times*,* he’s actually sat in the chair.”* (ID26; Parent, 10–14 and 15–19).

*“I think it’s just nice. She’s [the dentist] just nice to everyone… She gives you stickers when you finish… She always makes sure that you’re okay with the feeling going in… I like the whole place.”* (ID18; CYP, 10–14).

Negative experiences often arose from sensory overload, misunderstanding of autistic behaviours, or rushed communication. DHPs observed that such experiences could profoundly affect a child’s willingness to attend future appointments.

*“…the only thing I can remember from going to the dentist is… My teeth needed to come out… They put mint on my teeth and I hate mint… They destroyed the gum that was keeping it there. It was very painful. They said it wasn’t going to hurt.”* (ID29; CYP 10–15).

#### Impact of negative experiences on future access

Traumatic experiences created lasting barriers to care. CYP described anticipatory anxiety and fear of appointments, while parents explained that rebuilding trust required considerable effort:

*“…she’s saying*,* ‘It was traumatising. It was a nightmare.’… [To us] it was a tiny incident*,* to her it’s massive. It’s changed her whole perspective… she hasn’t been since.”* (ID27; Parent, 10–14 and 15–19).

## Theme 2: access to dental care by autistic CYP

### Service limitations

Families reported challenges accessing NHS dental care, including difficulty finding practices accepting new patients, long waiting times, and cancelled appointments.

DHPs also recognised systemic barriers:

*“Lots of parents know it’s important*,* [but] they can’t access a dentist because practices aren’t taking new patients.”* (DID3; DHP).

Parents further highlighted the lack of clear information about which practices were autism-friendly or equipped to meet their child’s needs, suggesting that such guidance should be provided upon diagnosis to help families navigate the system more effectively.

“*Trying to get the little one a dentist. That’s been extremely hard. He has to be seen by a normal dentist to assess him [for referral] but he won’t cooperate…nobody wants to take him on so we can’t get a referral*.” (ID16; Parent, 10–14).

#### Lack of choice and its consequences

Limited choice often forced families to remain with dentists who did not meet their child’s needs, leading to treatment refusal or heightened anxiety. Parents described feeling “stuck” with providers who did not connect well with their child, unable to change due to the shortage of NHS dental places. This lack of agency left families feeling powerless and, in some cases, led to disengagement from dental care altogether, with children sometimes refusing treatment even when in pain.

#### Private care as a partial solution

Some families chose private care to maintain continuity or access a dentist better suited to their child’s needs. While this mitigated some access challenges, financial burden was a concern. Some parents and carers were able to absorb the financial impact to access private care, even when it cost a substantial amount each month. Others, however, could not afford this option and expressed concern that the current system risks creating a “two-tier” model, where only those with financial means can access autism-sensitive dental care.

### Theme 3: CYP-related factors influencing dental experience and access

#### Sensory sensitivities

Autistic CYP reported that bright lights, loud sounds, unfamiliar smells, and tactile sensations could be overwhelming. Parents highlighted that these sensory challenges often compounded one another, while DHPs acknowledged that such sensory sensitivities could create significant barriers to accessing care:

“*Horrible… I think it might have been the bright lights and like all the machinery that was there. … it was like being a closed room*,* like we were – not trapped but like enclosed.*” (ID4; CYP, 15–19).

Parents/carers spoke about the “*stacking*” of sensory challenges and how these can compound to make the experience unbearable.

*“[My daughter] copes with sensory difficulties but they stack… if we’d come in and something had happened at school and then we had to wait… All of these things stack*,* so it can reach a point where then a lighting level that would normally be okay is intolerable. It just causes a panic attack.”* (ID5; Parent, 15–19).

CYP suggested several ways to improve the sensory experience in dental settings, including soundproofing, providing videos with headphones, creating separate waiting areas for children, using softer colours, minimising background noise, reducing the use of loud tools, avoiding strong-tasting products, dimming lights, and offering dark glasses.

#### Changes in routine and unfamiliar environments

Disruptions to routines or exposure to new environments caused distress and could lead to refusal of treatment. For some CYP, even travelling to the dental clinic or entering the waiting room could be overwhelming, particularly if the space was noisy or crowded.

*“…I had to leave school but not at the right time… I started crying*,* I didn’t like it…”* (ID28; CYP, 5–9).

CYP and parents emphasised the benefits of preparatory tools such as video tours or social stories.

#### Anxiety and fear of the dentist

Fear of the unknown, negative past experiences, and external influences, such as peers’ stories or media portrayals, contributed to children’s anxiety about dental visits.

“*No*,* I don’t like the dentist cos when you go they rip out all of your teeth and put in like big gold ones instead and it really hurts*” (ID38, CYP 5–9).

“…*she was shaking and crying and I had her hand… The anxiety starts as soon as the dentist says*,* ‘Open your mouth’* …” (ID34; Parent, 10–14).

Parental anxiety often transferred to the child:

*“Parents often come with their own anxieties which obviously kids pick up on… which isn’t helpful.”* (DID10; DHP).

Some children described feeling scared or distressed during treatment:

*“I’m scared-it’s the typically scary thing. Fear of the unknown really.”* (ID12; CYP, 10–14).

All three participant groups (CYP, parents/carers, and DHPs) agreed that helping children familiarise themselves with the dental environment, through visits, practice at home, or comfort objects, can reduce stress and improve future experiences.

“*These familiarisation sessions take time … However*,* it really helps manage the children’s and parents’ anxieties. If you can start right in the beginning the future looks much better.*” (DID2; DHP).

#### Transition to adulthood and executive functioning

Young people expressed concerns about independently managing appointments and oral health:

“*I’m no good at organising things and answering phone calls on my own… I usually let Mum and Dad do the complex organising for me…”* (ID7; CYP, 15–19).

Parents and carers also expressed concerns about their children’s ability to manage these tasks and felt that, during this transition period, they still needed to act as advocates for their child.

### Theme 4: provider-related factors affecting access

#### Awareness and understanding of autism among DHP

Families valued DHPs who recognised autism and adapted care accordingly. They highlighted that autism can be a “hidden disability” and that children may be “good at masking,” which can lead to challenges being underestimated. Lack of firsthand experience could result in misinterpretation of behaviours:

*“I think you have some dentists that aren’t aware… unless your child conforms to what they’ve read*,* they don’t get it because they’ve not interacted with that.”* (ID27; Parent, 5–9 and 10–14).

Parents and carers also described occasions when their child was distressed in the waiting room or clinical space, feeling criticised for not adequately “controlling” their child.

*“It was mainly the attitude of the reception staff… I think they just didn’t have that understanding of people’s needs… In the end I said*,* ‘Look*,* I’m sorry. We’re going to have to leave.’ [Daughter] was really distressed… She probably hadn’t had any training about what people might need.”* (ID1; Parent, 5–9).

#### Communication

Clear, patient, and direct communication was essential, especially for children needing extra processing time. Parents and DHPs emphasised adapting to each child’s preferred communication style and using language suited to their age and abilities.

CYP emphasised the importance of dental team members speaking directly to them and explaining exactly what they were going to do. Parents/carers highlighted the need for DHPs to learn each child’s preferred communication style and to use clear, unambiguous language appropriate to the child’s age, knowledge, preferences, and abilities. Community and specialist DHPs highlighted the importance of giving CYP processing time, with pauses and opportunities to reflect on any concerns or confusions. Children noted difficulties understanding dentists with strong accents:

*“…Accents are terrifying for me…”* (ID12; CYP, 15–19).

Parents and DHPs suggested that strategies such as repeating instructions slowly, using visual aids, and confirming understanding could help children follow conversations and feel more confident when practitioners have diverse accents or communication styles.

Where children are non-speaking, parents/carers may bring them to see the dentist because of behavioural changes that may indicate pain. DHPs reported challenges when autistic children have limited awareness or words to describe pain:

*“…it is hard because you can’t always understand…the first thing you always think of*,* “Behaviour’s changed – are they in pain?”…so it’s about having a look in*,* and just talking to them as I am talking to you but you know you’re not going to get anything back…”* (DID11; DHP).

#### Trust and rapport

Continuity and relationship-building were essential. Disruptions in care or negative experiences could undermine trust in both clinicians and the system. DHPs emphasised the importance of connecting with the child and family:

“…*it’s all about having the confidence of the patient in you and the family. Because*,* it’s a team approach.”* (DID8; DHP).

Parents highlighted the value of stability and seeing the same clinician:

*“…when they go to the dentist*,* they need to have stability and they need to just know that they’re going to see that same [person]…* (ID3; Parent, 15–19).

#### Parent/carer advocacy

Parents/ carers felt it was important for the dentist to enable them to feel empowered to advocate for their child and to ensure their views were heard. As one mother stated, “*when it comes to my kids*,* I’m the authority*.” However, while some parents and carers were comfortable advocating for their child with DHPs, others were hesitant, fearing they might offend the professionals or felt they lacked authority. DHP highlighted the importance of reducing hierarchical barriers and involving both children and their parents or carers in decision-making.

#### Flexibility and time constraints

Time pressures in NHS dentistry limited flexibility, which could increase stress for CYP, parents, and providers.

*“… and you just*,* as a parent*,* you felt like the dentist was looking at you like “Sort this out. I’ve got another patient coming in.” Felt very hurried which didn’t help*.” (ID9; Parent, 10–14).

Extended preparation and familiarisation were difficult to accommodate within standard appointments:

*“…Under the UDA treadmill I have 10 minutes. For an autistic child*,* it can take them 10 minutes to come from the waiting room into the surgery*,* screaming…”* (DID14; DHP).

Both parents/carers and CYP said that keeping appointments to time would help make dental visits more successful particularly for CYP who are very time-oriented and whose anxieties are exacerbated by waiting.

*“… so if her appointment’s at 2:20*,* she wants to be seen at 2:20. So*,* anything after she becomes really agitated and wants to go – she gives him like a time limit and says*,* “Right. You’ve got five minutes. If you haven’t come out and called me*,* then I’m going.”* (ID21; Parent, 10–15).

Parents and carers suggested strategies to reduce waiting times, including phoning the clinic before arrival, allowing children to wait in the car until called, and scheduling appointments during quieter periods. They also noted that keeping appointments on time helped children who are very time-oriented and whose anxieties increase with waiting.

### Theme 5: organisational changes and training to improve access to dental care for autistic CYP

#### Improving access

DHPs suggested ways to improve access to NHS dental care for autistic CYP. These included: facilitating appropriate referrals and reducing delays by providing primary care dentists with clearer criteria for referral; raising awareness among healthcare and other professionals about the range of dental care services available and how to refer or signpost; adjusting NHS contracts to allow paid referrals for CYP to see dental hygienists or therapists (participants did not specifically mention specialists in Paediatric Dentistry or Special Care Dentistry, likely reflecting local availability or awareness of referral pathways); and ensuring DHPs are trained in, and facilities available for, intravenous sedation as an alternative to general anaesthetic to enable less radical treatment.

#### Sharing information

Parents and carers highlighted that medical questionnaires do not currently allow them to declare their child as autistic, while dentists reported receiving insufficient information from referrals. Recording children’s preferences, communication needs, sensitivities, and triggers was seen as vital for tailored care. Dental passports were suggested as a tool to improve information sharing between providers.

#### Training needs

DHPs emphasised the need for autism-focused training to improve awareness and communication. Calls were made for continued professional development (CPD) co-designed with the autism community, resources for secondary care dentists, and training in behaviour management techniques for primary care teams. Increasing autism and special care dentistry training within dental school curricula was also recommended.

### Theme 6: key messages for dental students

On the suggestion of CYP in our research advisory group, all study participants were asked what key messages, about caring for autistic CYP, they would give to dental students just about to start out on their careers. Participants suggested that dental students and new graduates should receive training on autism awareness, sensory considerations, and effective communication strategies. They emphasised the importance of empathy, patience, and continuity to build trust with autistic CYP and their families. Exposure to community and special-care settings was viewed as valuable for developing confidence and understanding in working with neurodiverse populations. Their responses are given in Table 4.

**Table 4:** Advice on caring for autistic children and young people for dental students starting out on their career [from CYP (C), parents/carers (P) and dental health professionals (D)]

Training	Get some training to increase their knowledge and understanding of autism (All) Take any opportunities to spend time in a special care dentistry setting (D) Understand how autism can present differently in different individuals (All) Find out how autism can affect children in cleaning their teeth (P) Learn how to treat autistic CYP in your role as dentist (P) Learn what additional support they might need. (P) Learn communication techniques e.g. PECs, Makaton (P, D)
Awareness	Be aware girls can be autistic too and that they may present quite differently to boys (C, P) Be aware of masking (P) Don’t make assumptions based on autism tropes (D) Be aware of the range of sensory sensitivities and “stacking” (P)
Attitude	Be kind (C)
Give time	Be patient (C, P) Give more time (P, D)
Communication	Give patients time to process (C, P) Take time to listen; make sure the individual and family feel listened to (P, D) Be specific in your language and don’t joke (C, P) Read the non-verbal clues (P) Take time to introduce yourself and your team (without masks) (C, P) Use different ways of communication to suit the child (e.g. pictures) (ALL)
Get to know the child and their family	Be guided by the child (P) Learn individual likes and dislikes including sensory sensitivities and triggers (P) Find out what works for that individual family (D) Have a profile of the patient that can share with other DCP (P)
Create a positive atmosphere	Be empathic (D) Be reassuring (D) Create points of connection – toys, interests (P) Focus on what might work rather than the negatives (P) Don’t tell children off (C)
Build trust	Take time to build trust through familiarisation visits (P) Work with the patient & their family (D)
Explain	Explain what you are going to do before doing it, how and why using simple non-medical language (ALL) Explain how what you are doing will feel (D) Tell children and their parents in detail how to brush their teeth (C)
Be flexible	Be creative and responsive in approach; think outside the box (D)
Think about the environment	Make sure that posters aren’t potentially frightening or could raise anxiety (C)
Carrying out the examination	Be gentle (C) Always ask first before doing something (C)
Time management	Keep waiting times to a minimum (C, P) Complete procedure as quickly as possible (C)
Referrals	Make timely referrals (C)

*C *Children, *P *Parents/Carers, *D *Dental Health Professionals, *PECs *Picture Exchange Communication System

### Summary of key insights-understanding the interplay of factors affecting dental care for autistic CYP

This series of interviews with autistic CYP, parents/carers, and DHPs highlights the complex, interrelated factors shaping autistic CYP’s access to and experiences of dental care. Autistic CYP experience sensory and communication barriers; parents acted as advocates within constrained systems; and DHPs struggled to deliver flexible, person-centred care within structural limits.

## Discussion

### Main findings

This qualitative study explored the factors influencing autistic CYP’s access to dental care in Southwest England, incorporating perspectives from CYP, parents/carers, and DHPs. The findings demonstrate that autistic CYP’s experiences at the dentist are shaped by a complex interplay of child-related, provider-related, and system-level factors reflecting the dynamic interplay between individual neurodiversity and service structures. Sensory sensitivities, changes in routine, and anxiety often interact, creating significant barriers to attending appointments and engaging with care. These child-related challenges are compounded when access to appropriate dental services is limited, such as when families encounter long waiting times, a lack of choice in providers, or difficulties navigating referrals. Provider factors, including awareness of autism, communication skills, and the ability to build trust, further influence how these challenges manifest. For example, a dentist who understands a child’s sensory needs and communicates clearly can mitigate anxiety and facilitate cooperation, whereas lack of understanding can reinforce fear and avoidance. Similarly, consistent relationships with dental teams, combined with parental advocacy and tailored preparation, can buffer against systemic limitations and negative experiences. This interconnected perspective highlights that improving dental care for autistic CYP requires coordinated attention to the child’s needs, provider training, and organisational support, rather than isolated interventions in any single domain.

### Interpretation of findings

The sensory sensitivities, anxiety, and communication differences described by participants echo findings from Chauhan et al. [6], who portrayed autistic children’s dental experiences as “an explosion in the mouth.” However, the present study extends understanding by identifying how these personal experiences intersect with systemic barriers such as NHS contract limitations and limited training opportunities.

Clear, patient, and adaptable communication emerged as a critical factor, particularly for children who require additional processing time. Children reported difficulties understanding dental professionals with strong accents, which could increase anxiety and reduce engagement. Parents and DHPs suggested practical strategies such as using visual aids, repeating instructions slowly, or asking children to confirm understanding to help overcome these barriers, highlighting the need for training that supports communication across diverse linguistic backgrounds.

Our results support earlier research highlighting parental advocacy as a critical facilitator of access [13] yet al.so expose the emotional burden parents bear in navigating inflexible systems. The present study adds new insights into the transition to adulthood, where executive-functioning challenges impede continuity of care, underscoring the need for tailored support during this critical developmental stage.

At the professional level, DHPs expressed a genuine desire to provide inclusive care but were constrained by time, resources, and policy frameworks. This tension mirrors broader concerns regarding the sustainability of NHS dentistry [26] and the equity of access for people with additional needs.

Together, these findings underscore that improving access for autistic CYP requires more than clinical adaptation. It calls for systemic change to ensure adequate time, training, and flexibility within NHS dental care.

### Strengths and limitations

A key strength of this study lies in its co-produced design and the active involvement of autistic CYP as contributors rather than passive participants. Their participation provided valuable insights into communication preferences, sensory triggers, and coping strategies, ensuring that findings authentically reflected participants’ perspectives and experiences.

The inclusion of multiple stakeholder groups, CYP, parents/carers, and DHPs, enabled triangulation and enriched understanding of the diverse perspectives influencing access to dental care. By employing both inductive and deductive coding, the analysis effectively integrated participants’ experiences with existing theoretical frameworks of healthcare access [7, 11], situating findings within broader conceptual models while remaining grounded in participants’ realities.

To enhance trustworthiness, strategies such as triangulation, member checking, thick description, reflexive journaling, and audit trails were employed, strengthening the credibility, dependability, confirmability, and transferability of the findings [24, 25].

The research team, trained in dentistry and public health, recognised that professional assumptions might influence interpretation. Continuous reflexive dialogue and the inclusion of autistic advisory members (TV and IV) helped mitigate potential bias and ensured the authentic representation of autistic voices throughout the research process.

A limitation of this study is the challenge of collecting credible qualitative data from children due to variation in linguistic, cognitive, and communicative abilities. While children as young as five can articulate their views [27], children aged four to eleven may have limited capacity for abstract reflection and sustained verbal reasoning, resulting in wide variability in the depth and focus of responses [28, 29]. This was mitigated through age- and ability-adapted interview guides and triangulation with parent/carer and dental professional data, strengthening the credibility of the themes.

Other limitations include the sample being drawn from a single region, with many participants connected to autism-support networks, which may restrict transferability. Data collection occurred during a period of reduced NHS dental service availability following the COVID-19 pandemic, which could have amplified access difficulties.

### Implications for policy, practice, and education

The findings have several implications. First, autism-informed training for the dental workforce is essential. DHPs require not only theoretical understanding but also practical strategies, delivered through co-produced training with autistic individuals and families, to enhance confidence and competence. Second, flexible appointment structures and adjusted NHS remuneration models could allow more time for acclimatisation and communication support. Third, improved information-sharing mechanisms, such as “dental passports” or pre-visit forms documenting communication and sensory preferences, could prevent repeated distress and reduce reliance on parental advocacy.

In dental education, embedding neurodiversity awareness and communication skills within undergraduate and continuing professional development (CPD) curricula could cultivate empathy and preparedness among future practitioners [30]. Clinical placements in special-care dentistry and interprofessional learning may further reinforce these competencies.

### Future research

Future research should evaluate autism-informed training programmes and investigate long-term outcomes of service modifications such as sensory-adapted environments or extended appointment models. Longitudinal studies could explore how autistic CYP navigate the transition to adult dental care, while participatory approaches should continue to prioritise autistic voices in co-designing interventions.

## Conclusions

Autistic CYP face distinct and often preventable barriers to dental care rooted in sensory, communicative, and systemic factors. By integrating the perspectives of CYP, parents, and professionals, this study demonstrates that equitable access requires more than clinical adaptation. It highlights the significance of structural flexibility, empathy, and genuine partnership. Co-produced solutions and autism-informed education are vital for creating inclusive, compassionate dental services that meet the needs of neurodiverse children and young people.

## Supplementary Information

Additional file 1: Interview guides.

## Data Availability

The datasets generated and/or analysed during the current study are not publicly available due to the sensitive nature of interview transcripts related to a vulnerable population group. De-identified excerpts are available from the corresponding author on reasonable request.

## Abbreviations

COREQ: Consolidated criteria for reporting qualitative research
CYP: Children and young people
DHP: Dental health professionals
NHS: National health service
OH: Oral health
